# Cariprazine as a useful treatment for patients with schizophrenia and antipsychotic-induced extrapyramidal symptoms: a case report and literature review

**DOI:** 10.1192/j.eurpsy.2022.2047

**Published:** 2022-09-01

**Authors:** C. Llach, L. Ilzarbe, M. Sagué-Vilavella, N. Arbelo, R. Catalan, F. Gutierrez

**Affiliations:** Hospital Clinic de Barcelona, Department Of Psychiatry And Psychology, Institute Of Neuroscience, Barcelona, Spain

**Keywords:** cariprazine, schizophrénia, extra-pyramidal syndrome, negative symptoms

## Abstract

**Introduction:**

The discovery of second-generation antipsychotics represented an authentic breakthrough for the management of psychotic disorders. Nevertheless, they still don’t adequately manage some aspects of these disorders, such as negative symptoms (NS), cognitive impairment, or extra-pyramidal symptoms (EPS). New-generation antipsychotics present different pharmacological mechanisms and have been reported to ameliorate these aspects. Among them, cariprazine acts as a D2/D3 partial agonist with variable affinity with serotoninergic receptors, and many studies show its efficacy for preventing and treating positive symptoms as well as for the management of NS and EPS.

**Objectives:**

To report a case of a patient diagnosed with schizophrenia with highly invalidating antipsychotic-induced EPS that remitted after switching to cariprazine, while maintaining clinical stability. To review literature about cariprazine and its relationship with NS and EPS.

**Methods:**

We describe the case of a 66-year-old woman diagnosed with schizophrenia and under treatment with three-month injectable paliperidone 175mg. During her follow-up at outpatient clinic, she presented a progressively highly invalidating non-trembling parkinsonian syndrome attributable to medication. Paliperidone plasmatic levels were within therapeutic range. An antipsychotic switch was agreed, and cariprazine was started.

**Results:**

The switch from a second-generation antipsychotic to cariprazine entailed the remission of a highly invalidating EPS while improving some of the NS and maintaining psychopathological stability.

**Conclusions:**

Assessing and differentiating NS and EPS is of an utmost importance during the follow-up of patients under antipsychotic treatment. Cariprazine is an interesting alternative when treating patients diagnosed with psychotic disorders that present mostly NS and antipsychotic-induced EPS.

**Disclosure:**

No significant relationships.

